# Transcriptomics analysis of ethanol treatment of male *Aedes aegypti* reveals a small set of putative radioprotective genes

**DOI:** 10.3389/fphys.2023.1120408

**Published:** 2023-01-30

**Authors:** Matthew Pinch, Harley Bendzus-Mendoza, Immo A. Hansen

**Affiliations:** ^1^ Department of Biology, New Mexico State University, Las Cruces, NM, United States; ^2^ Department of Computer Science, New Mexico State University, Las Cruces, NM, United States; ^3^ Institute of Applied Biosciences, New Mexico State University, Las Cruces, NM, United States

**Keywords:** *Aedes aegypti*, sterile insect technique, radioprotection, transcriptomics, ionizing radiation

## Abstract

**Introduction:** Sterile Insect Technique (SIT) is based on releasing sterilized male insects into wild insect populations to compete for mating with wild females. Wild females mated with sterile males will produce inviable eggs, leading to a decline in population of that insect species. Sterilization with ionizing radiation (x-rays) is a commonly used mechanism for sterilization of males. Since irradiation can cause damage to both, somatic and germ cells, and can severely reduce the competitiveness of sterilized males relative to wild males, means to minimize the detrimental effects of radiation are required to produce sterile, competitive males for release. In an earlier study, we identified ethanol as a functional radioprotector in mosquitoes.

**Methods:** Here, we used Illumina RNA-seq to profile changes in gene expression of male *Aedes aegypti* mosquitoes fed on 5% ethanol for 48 hours prior to receiving a sterilizing x-ray dose, compared to males fed on water prior to sterilization.

**Results:** RNA-seq revealed a robust activation of DNA repair genes in both ethanol-fed and water-fed males after irradiation, but surprisingly few differences in gene expression between ethanol-fed and water-fed males regardless of radiation treatment.

**Discussion:** While differences in gene expression due to ethanol exposure were minimal, we identified a small group of genes that may prime ethanol-fed mosquitoes for improved survivability in response to sterilizing radiation.

## 1 Introduction

As more evidence emerges to demonstrate the spread of insects with evolved resistance to many commonly used chemical agents, novel mechanisms are necessary to prevent the spread of insect pests (Liu et al., 2000; Moyes et al., 2017; Amelia-Yap et al., 2018; Fotakis et al., 2018; Macoris et al., 2018; Chen et al., 2019; Kandel et al., 2019; Ngongang-Yipmo et al., 2022; Pichler et al., 2022; Pudasaini et al., 2022). Sterile Insect Technique (SIT) is an effective biological control mechanism that has been successfully used to eliminate populations of a variety of insect pest species (Vargas-Terán et al., 1994; Vreysen et al., 2000; Ant et al., 2012; Hyseni et al., 2012). The practice of SIT dates to 1954, when the process was first implemented to control the screw-worm fly (*Cochliomyia hominivorax*), a major livestock pest in the Americas (Bushland et al., 1955; Knipling, 1959; 1960). From the 1950s into the 1990s, SIT was used to eradicate *C. hominivorax* from the southern United States and much of Central America and the Caribbean (Vargas-Terán et al., 2021), demonstrating its effectiveness as an alternative insect control mechanism to use of chemical insecticides. Since that time, SIT has been used to successfully control populations of other agricultural pests, and novel biological control mechanisms including RIDL (Release of Insects carrying a Dominant Lethal gene) have been developed (Yen and Barr, 1971; 1973; Alphey, 2002; Alphey and Andreasen, 2002; Alphey et al., 2002; Atyame et al., 2011; Killeen et al., 2013; Beebe et al., 2021). A common principle underlying these biological control strategies is the mass-release of laboratory-reared male insects to compete for mating with wild females. In these techniques, matings involving these males should produce inviable embryos, leading to a swift decline in population of the pest insect in the treated area.

SIT represents an attractive option for insect control, as it does not require genetic manipulation (RIDL) or crossing of insects bearing different strains of *Wolbachia* (cytoplasmic incompatibility). Rather, SIT only requires treatment of male insects to a non-lethal dose of ionizing radiation to sterilize their gametes (Knipling, 1955; 1959; Bakri et al., 2021). When planning a SIT treatment, the radiation dosage should be high enough to cause a single dominant lethal mutation in every single sperm cell (Robinson, 2021). While insects are notoriously resistant to radiation (Cole et al., 1959; Ross and Cochran, 1963), high dosages of radiation can produce toxic effects not only in the sensitive germline cells but also in somatic cells. Reactive oxygen species (ROS) created by ionizing radiation and subsequent mutations in somatic cells can reduce SIT male fitness relative to the wild males that they will need to compete with. Therefore, successful SIT implementation requires management or amelioration of the harmful side effects of radiation exposure while still creating sterile males.

The yellow fever mosquito (*Aedes aegypti*) is a vector of many deadly diseases. Due to evolution of insecticide resistance and global climate change, these mosquitoes have rapidly expanded their home range out of the tropical regions of the world into more temperate areas including the western United States and parts of Europe (Kraemer et al., 2015; Kandel et al., 2019), placing larger portions of humanity at risk of contracting the diseases spread by this vector. Knowing this, it is of vital importance to implement novel means of controlling the spread of *Ae. aegypti* in addition to use of traditional chemical pesticides. Large-scale releases of RIDL males in different locations around the world have provided promising evidence that biological control mechanisms can be a useful tool for eradicating populations of *Ae. aegypti* (Harris et al., 2011; Harris et al., 2012; Lacroix et al., 2012; Carvalho et al., 2015), but local communities have previously been hesitant to allow the mass release of genetically modified mosquitoes in their vicinity (Maxmen, 2012; Waltz, 2021). SIT offers a viable alternative for the biological control of *Ae. aegypti* as no genetic manipulations of mosquitoes is required in the mass production of released males. A previous study from our laboratory has demonstrated that low doses of dietary ethanol can provide a radioprotective effect for male *Ae. aegypti* exposed to sterilizing doses of ionizing radiation, increasing their survival and fitness without reducing sterilization efficiency (Rodriguez et al., 2013). The genetic and physiological mechanisms underlying this observed radioprotective effect are not known but may be due to priming by increased oxidative stress caused by ethanol (Puddey et al., 1998; Wu and Cederbaum, 2003; Albano, 2006). A comprehensive study of gene expression in male *Ae. aegypti* treated with low amounts of ethanol prior to irradiation will provide great insight into how ethanol may prime mosquito cells to better survive ionizing radiation, and may allow for identification of even more effective radioprotector compounds for use in future radiation-based SIT programs.

Here, we describe the effects of ethanol feeding and ionizing radiation on gene expression in male *Ae. aegypti* and report a set of genes with increased expression after ethanol-feeding that may “prime” male mosquito fitness to subsequent sterilizing doses of ionizing radiation. The results of this experiment provide important insights into how ethanol may prime organisms to cope with radiation exposure and inform the designs of future SIT-based mosquito control programs.

## 2 Materials and methods

### 2.1 Mosquito rearing

*Aedes aegypti* Liverpool strain mosquitoes were used for this study. The Liverpool strain used in this study was obtained from BEI Resources (Molestina, 2010), and maintained for at least four generations in our insectary at the Molecular Vector Physiology Laboratory prior to use. Eggs were hatched in pans containing 1L of deionized water. Larvae were reared in groups of approximately 250 per pan at 37°C. Larvae were fed Special Kitty cat food pellets (Walmart Stores Inc., Bentonville AR) *ad libitum*. Pupae were separated into dishes and placed into large (30 × 30 × 30 cm) BugDorm-1 cages and allowed to emerge under standard conditions (27°C, 80% humidity, 14 h: 10 h light:dark cycle) with a 20% sucrose solution as a nutrient source. Approximately 24 h post-eclosion, mosquitoes were separated into new cages and reared for an additional 24 h with 20% sucrose solutions to feed on *ad libitum*. After this second 24 h period, sucrose solutions for half of the mosquitoes were replaced with deionized (DI) water, and sucrose solutions for the other half of mosquitoes was replaced with a 5% (v/v) ethanol solution (Rodriguez et al., 2013). Mosquitoes were reared for another 48 h on DI water or the 5% ethanol solution prior to irradiation.

### 2.2 Irradiation

Four groups of 30 male mosquitoes from each treatment (DI water or 5% ethanol) received a 50 gray dose of radiation in a Faxitron MultiRad 350 x-ray cabinet (Faxitron Bioptics, LLC, Tucson, AZ). A recent study used several radiation doses on adult male *Ae. aegypti*, including a comparable dose of 55 Gy, showed significant reduction in fertility with minor effects on male fitness (Somada et al., 2022), which provides support for our selection of 50 Gy as a sterilizing radiation dose in this study. Another four groups of thirty male mosquitoes from each treatment were placed into the x-ray cabinet for an equivalent amount of time as the irradiated mosquitoes, but the x-ray was not turned on. These groups served as controls.

After radiation or control treatment, all 16 groups were returned to their cages and allowed to recover for 24 h with 20% sucrose solutions. After these 24 h, mosquitoes were sampled for Illumina RNA-seq.

### 2.3 Total RNA isolation

Total RNA was isolated from each group of 30 male mosquitoes using a Qiagen RNeasy Mini extraction kit (Qiagen, Hilden, Germany). Briefly, each pool of mosquitoes was homogenized in 600 µL of lysis buffer using a VWR cordless motor (VWR, cat. No. 4774-370) and disposable polybutylene terephthalate pestles (VWR, cat. No. 4774-358). Lysed samples were centrifuged at 4°C and 10,000 × g for 3 min to pellet tissue. The supernatant was removed and mixed with an equal volume of 70% ethanol and applied to a RNeasy spin column and spun through at 4°C and 10,000 × g for 30 s. Once all RNA was bound to the columns, the columns were washed and treated with DNaseI (Qiagen, Hilden, Germany) for 15 min At room temperature to remove genomic DNA contamination following the RNeasy kit protocol. After DNaseI treatment, samples were washed and eluted in 50 µL of RNase-free water following the RNeasy kit instructions. RNA concentrations were measured using a NanoDrop One^C^ spectrometer (ThermoFisher, Waltham, MA) and samples were stored at −80°C prior to shipment to Azenta Life Sciences (South Plainfield, NJ) for library preparation and Illumina RNA-seq analysis.

### 2.4 Library preparation and illumina RNA-seq

Library preparation and Illumina RNA-seq were performed as previously described (Mitra et al., 2021). Briefly, RNA samples were quantified using a Qubit 2.0 Fluorometer (ThermoFisher Scientific, Waltham, MA, United States) and RNA integrity was checked using an Agilent TapeStation (Agilent Technologies, Palo Alto, CA, United States). RNA sequencing libraries were prepared using the NEBNext Ultra II RNA Library Prep Kit for Illumina using manufacturer’s instructions (New England Biolabs, Ipswich, MA, United States). Sequencing libraries were validated on the Agilent TapeStation (Agilent Technologies, Palo Alto, CA, United States), and quantified with a Qubit 2.0 Fluorometer (ThermoFisher Scientific, Waltham, MA, United States) and with quantitative PCR (KAPA Biosystems, Wilmington, MA, United States). Sequencing libraries were multiplexed and clustered onto a flowcell, loaded onto the Illumina HiSeq instrument and sequenced using a 2 × 150bp Paired End configuration. Image analysis and base calling were performed using the HiSeq Control Software. Raw sequence data (.bcl files) generated from Illumina HiSeq was converted into fastq files and de-multiplexed using Illumina bcl2fastq 2.20 software. One mis-match was allowed for index sequence identification.

### 2.5 Data analysis

Trimmomatic v.0.36 (Bolger et al., 2014) was used to trim, crop and remove adapter sequences from the sequence reads. Adapters were clipped using a custom fasta file created by combining the contents of the NexteraPE-PE. fa, TruSeq2-PE.fa, TruSeq2-SE.fa, TruSeq3-PE-2.fa, TruSeq3-PE.fa, and TruSeq3-SE.fa files bundled with Trimmomatic’s installation. Illuminaclip seed matches were set to 2, the palindrome clip was set to 30, and the simple clip was set to 10. Sliding window size was set to 5 bases, and the required quality was set to 30. Headcrop was set to 5 to remove the first 5 bases from the start of each read. To ensure that reads did not fall below 75 base pairs, Minlen was set to 75. Sequence read quality was assessed using FastQC v0.11.9. The trimmed reads were mapped to the most recent Ae. aegypti reference genome (AaegL5.3) found on Vectorbase using RSEM v1.3.1 (Li and Dewey, 2011). Transcript ID’s and their corresponding expression estimates (expected counts) were extracted from RSEM’s “gene.results” output file for each sample. Transcripts Per Kilobase Million (TPM) and Fragments Per Kilobase Million (FPKM) values were all generated by RSEM. Gene hit counts for each sample were collated into four different gene hit count tables based on treatment. Counts were then analyzed using the DESeq2 package (Love et al., 2014) in R (R Core Team, 2022). Genes with counts that summed to less than 10 across samples were removed from the table to exclude genes with very low expression or not expressed at all. To display the clustering of data between libraries, the raw counts were treated to a variance stabilizing transformation (VST) and a principal component analysis (PCA) using the plotPCA() function in the DESeq2 package. Using the pre-filtered raw count data and a generalized linear model (GLM) from the DESeq2 R Package, DEGs between each treatment group were examined. DESeq2’s built-in Benjamini Hochberg technique, used by default, was used to determine the adjusted *p*-values for the DEGs. The threshold for identifying differentially expressed transcripts was an adjusted *p*-value 0.05 and a log2 fold change of 2. Volcano plots visualizing genes within and without this threshold between samples were generated using the ggplot () and geom_point () functions from the ggplot2 package (Wickham, 2016) in R. The DESeq2 normalized gene counts and the pheatmap () function from the Pheatmap R package (Kolde, 2019) were used to create heatmaps to visualize the level of expression for the top 10 DEGs in each sample.

### 2.6 gene ontology analysis

The functions of the differentially expressed genes were determined by performing a gene ontology (GO) analysis. All DEGs and their associated Uniprot IDs were separated into 8 distinct groups: those significantly upregulated and those significantly downregulated within each group comparison. The associated Uniprot IDs for each of the 8 groups were used as the sample sets for the biological process analysis in the Panther database (Mi et al., 2019; Thomas et al., 2022). The Ae. aegypti reference proteome within the Panther database was used as the background set for each analysis. The threshold for significant gene ontology terms was an adjusted *p*-value (FDR) of 0.05 or less. The child terms for each significant GO term were then assessed for specific biological function.

## 3 Results

### 3.1 General RNA-seq results

**FIGURE 1:**
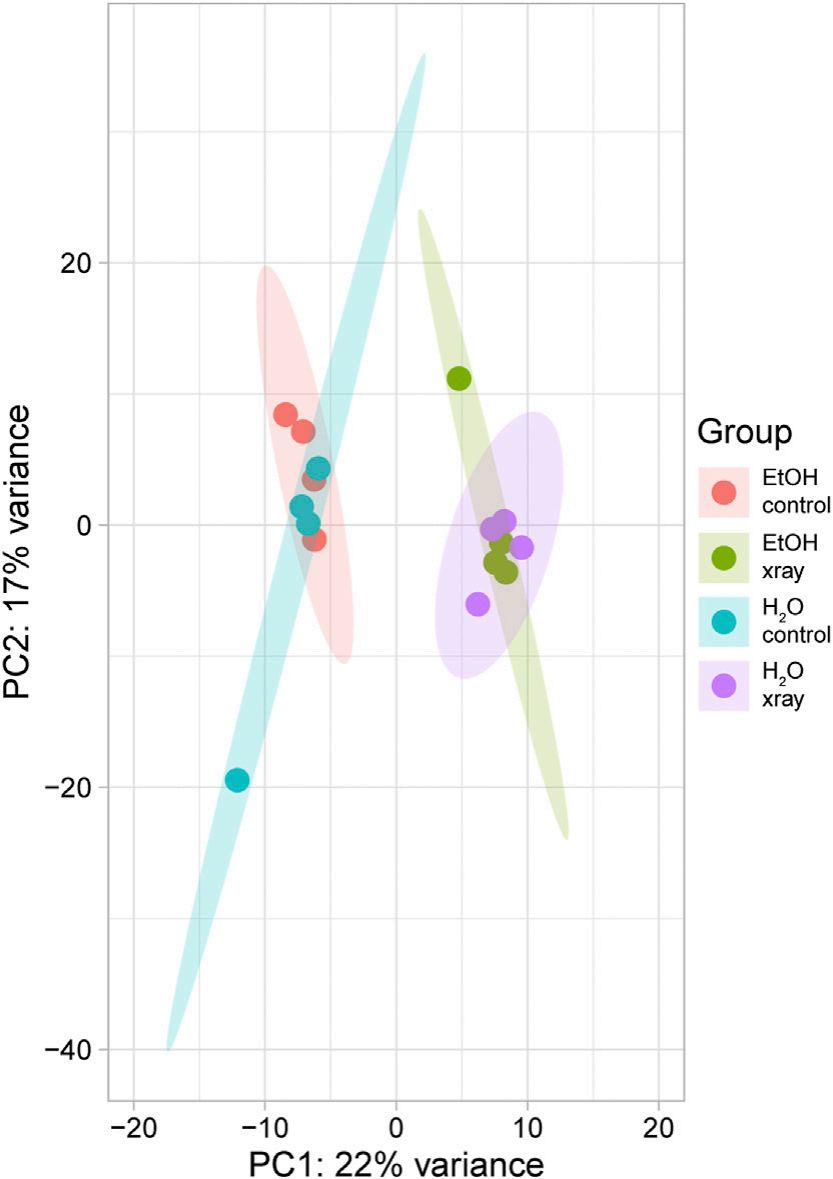
Principal component analysis of variability of gene expression in RNA-seq data from ethanol (EtOH) and water-fed non-irradiated (control) and irradiated (xray) mosquitoes. Points represent individual biological replicates (n = 4) from each treatment, and surrounding ovals represent 95% confidence intervals.

RNA-seq produced an average of 27,730,613 reads per sample (443,689,803 total reads) with an average yield of 8,319 Mbases (133,107 total Mbase yield) (Supplementary Table S1). The mean percent of bases with a quality score greater than 30, indicating a 99.9% confidence in call accuracy, was 91.25% with a low of 90.52% and a high of 91.71% (Supplementary Table S1). The mean quality score of our samples was 36.12 with a low of 35.42 and a high of 37.28 (Supplementary Table S1). After adapter trimming and removal of low-quality base pairs, an average of 14,820,086 reads per sample remained, of which an average of 11,763,177, or 80%, were mapped reads (Supplementary Table S1). Transcripts per million (TPM) values were calculated for all genes and these values were used for all subsequent analyses. Principal component analysis (PCA) of our RNA-seq data revealed minimal effects of feeding treatment on gene expression as ethanol and water-treated groups clustered closely together (Figure 1). Radiation treatment had a much larger effect on gene expression as non-irradiated and irradiated groups clustered separately from each other regardless of feeding treatment (Figure 1).

### 3.2 The effect of ethanol feeding on mosquitoes

**FIGURE 2:**
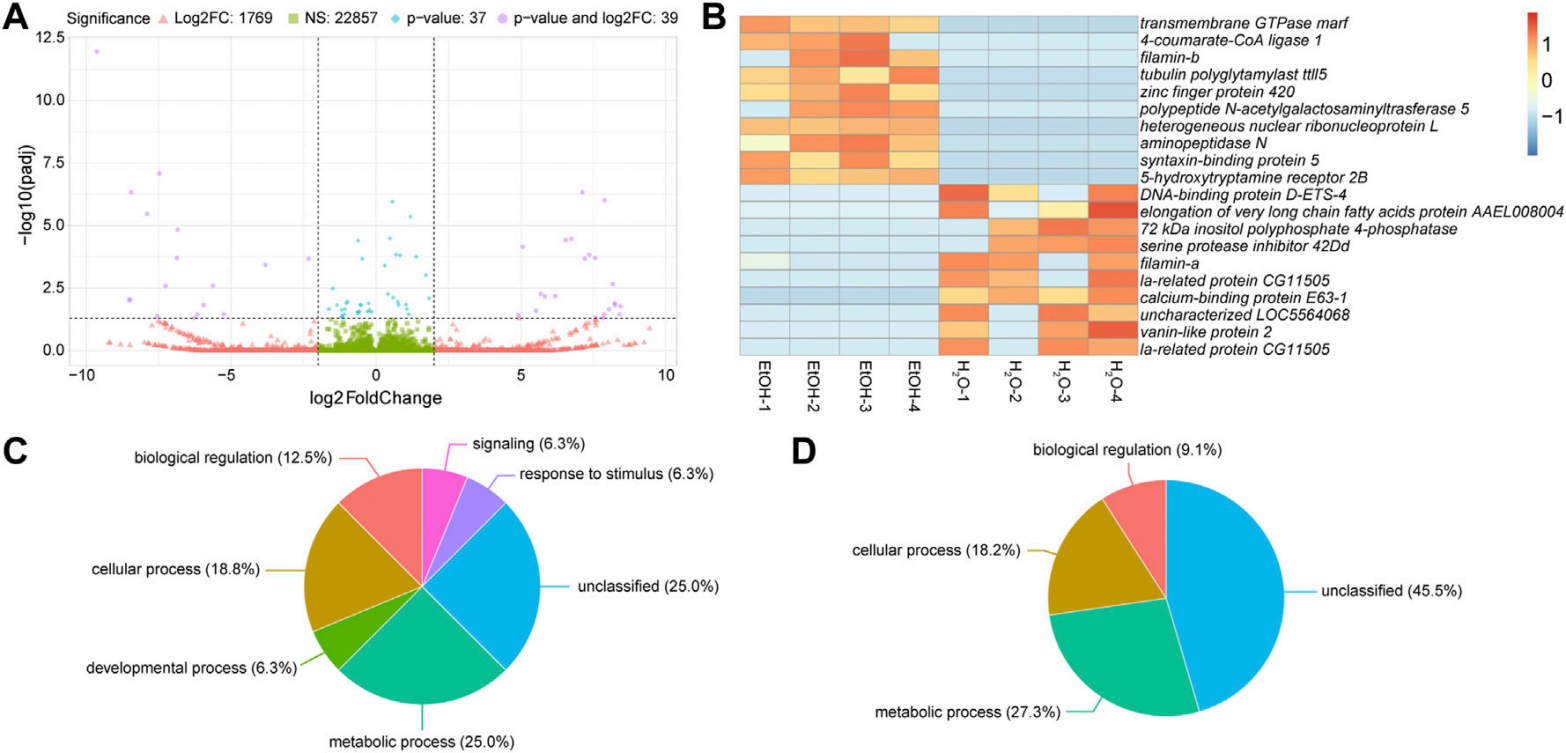
Differential gene expression between ethanol (EtOH) and water-fed non-irradiated mosquitoes. **(A)**. Volcano plot illustrating the differential expression patterns between transcripts with elevated expression in EtOH-fed mosquitoes (negative values) and elevated expression in water-fed mosquitoes (positive values). Vertical dashed lines represent four-fold change in expression, meaning any values more extreme than those lines are at least four-fold different between the two treatments. The horizontal dashed line represents a *p*-value of 0.05, so any points above this line represent genes with significantly different expression (*p* ≤ 0.05) between the two treatments. Green squares represent mean expression of transcripts less than four-fold different and not significantly different between treatments; red triangles represent mean expression of transcripts greater than four-fold different but not significantly different between treatments; blue diamonds represent mean expression of transcripts less than four-fold different but still significantly different between treatments; purple circles represent mean expression of transcripts that are both four-fold different and significantly differentially expressed between treatments. **(B)**. Heatmaps showing the ten most significantly differentially expressed transcripts with at least four-fold increase in ethanol-fed mosquitoes or water-fed mosquitoes. Each column represents a single biological replicate, and each row represents an individual transcript. **(C and D)**. Pie charts of gene ontology (GO) analyses of genes upregulated in water-fed mosquitoes relative to ethanol-fed mosquitoes **(C)** and upregulated in ethanol-fed mosquitoes relative to water-fed mosquitoes **(D)**. Gene IDs were converted to Uniprot IDs, and all converted Uniprot IDs were used for GO analysis. Parent GO terms are represented in these charts, and the percentages represent the percent of the analyzed genes that were grouped into the corresponding category.

We first analyzed the differences in gene expression between water-fed control mosquitoes and ethanol-treated ones to identify which transcripts changed expression in response to ethanol. Of the 24,702 transcripts detected in these two treatments, 22,857 were not significantly different, and were expressed within four-fold difference of each other (Figure 2A). A further 1,769 transcripts were greater than four-fold differentially expressed between the two treatments, but not significantly different (Figure 2B). Of the remaining 76 significantly differentially expressed genes (*p* ≤ 0.05) (compiled in), only 39 were also at least four-fold different between treatments (Figure 2A). The top ten upregulated transcripts in ethanol-fed non-irradiated and water-fed non-irradiated treatments are shown in Figure 2B (a full heatmap of all differentially expressed transcripts is presented in Supplementary Figure S1).

We performed gene ontology (GO) analysis on the differentially expressed genes (Figures 2C, D) and observed increases in the enrichment of genes associated with metabolic process and genes with no GO classification in ethanol-fed mosquitoes. However, with so few differentially expressed genes, GO analysis provided minimal GO terms for this comparison, so we chose to describe specific genes with differential expression between ethanol and water-fed males (Table 1).

**TABLE 1:** Top ten genes with significantly different gene expression between ethanol-fed and water-fed non-irradiated male *Ae. aegypti*.

Increased in ethanol-fed				
Vectorbase ID	Entrez gene ID	Name	log_2_Fold change	padj
AAEL004471-RB	5564882	transmembrane GTPase Marf	**9.638929527**	1.10E-12
AAEL002658-RC	5575571	4-coumarate--CoA ligase 1	**8.527933778**	0.009374724
AAEL019576-RH	5564736	filamin-B	**8.485340627**	0.008992832
AAEL027751-RB	5569462	tubulin polyglutamylase TTLL5	**8.452733961**	4.66E-07
AAEL019833-RAD	5566385	zinc finger protein 420	**7.902005223**	3.41E-06
AAEL019605-RE	5577563	polypeptide N-acetylgalactosaminyltransferase 5	**7.554905524**	0.041589535
AAEL025268-RK	5572228	heterogeneous nuclear ribonucleoprotein L	**7.479738471**	8.27E-08
AAEL020609-RA	5577668	aminopeptidase N	**7.262804467**	0.002554124
AAEL006948-RE	5568540	syntaxin-binding protein 5	**6.874577393**	0.00019742
AAEL026043-RC	5575639	5-hydroxytryptamine receptor 2B	**6.848515429**	1.44E-05

Increased in water-fed				
Vectorbase ID	Entrez Gene ID	Name	log_2_Fold Change	padj
AAEL005147-RC	5566047	DNA-binding protein D-ETS-4	**8.438009231**	0.016480686
AAEL020502-RA	5568917	elongation of very long chain fatty acids protein AAEL008004	**8.403355828**	0.035252413
AAEL001367-RB	5570350	72 kDa inositol polyphosphate 5-phosphatase	**8.254052481**	0.014817424
AAEL007765-RE	5569575	serine protease inhibitor 42Dd	**8.243355026**	0.012496176
AAEL023148-RE	5572167	filamin-A	**8.173864164**	0.002158609
AAEL015118-RT	5573995	la-related protein CG11505	**8.038036625**	0.022079605
AAEL012513-RU	5576419	calcium-binding protein E63-1	**7.889793019**	9.71E-07
AAEL025030-RA	5564068	uncharacterized LOC5564068	**7.869369901**	0.035252413
AAEL025718-RB	5566416	vanin-like protein 2	**7.826296519**	0.04855584
AAEL015118-RJ	5573995	la-related protein CG11505	**7.579830853**	0.04855584

log_2_Fold Change values represent the log_2_-normalized fold change in gene expression between ethanol-fed and water-fed mosquitoes.

### 3.3 The effect of radiation on mosquitoes

**FIGURE 3:**
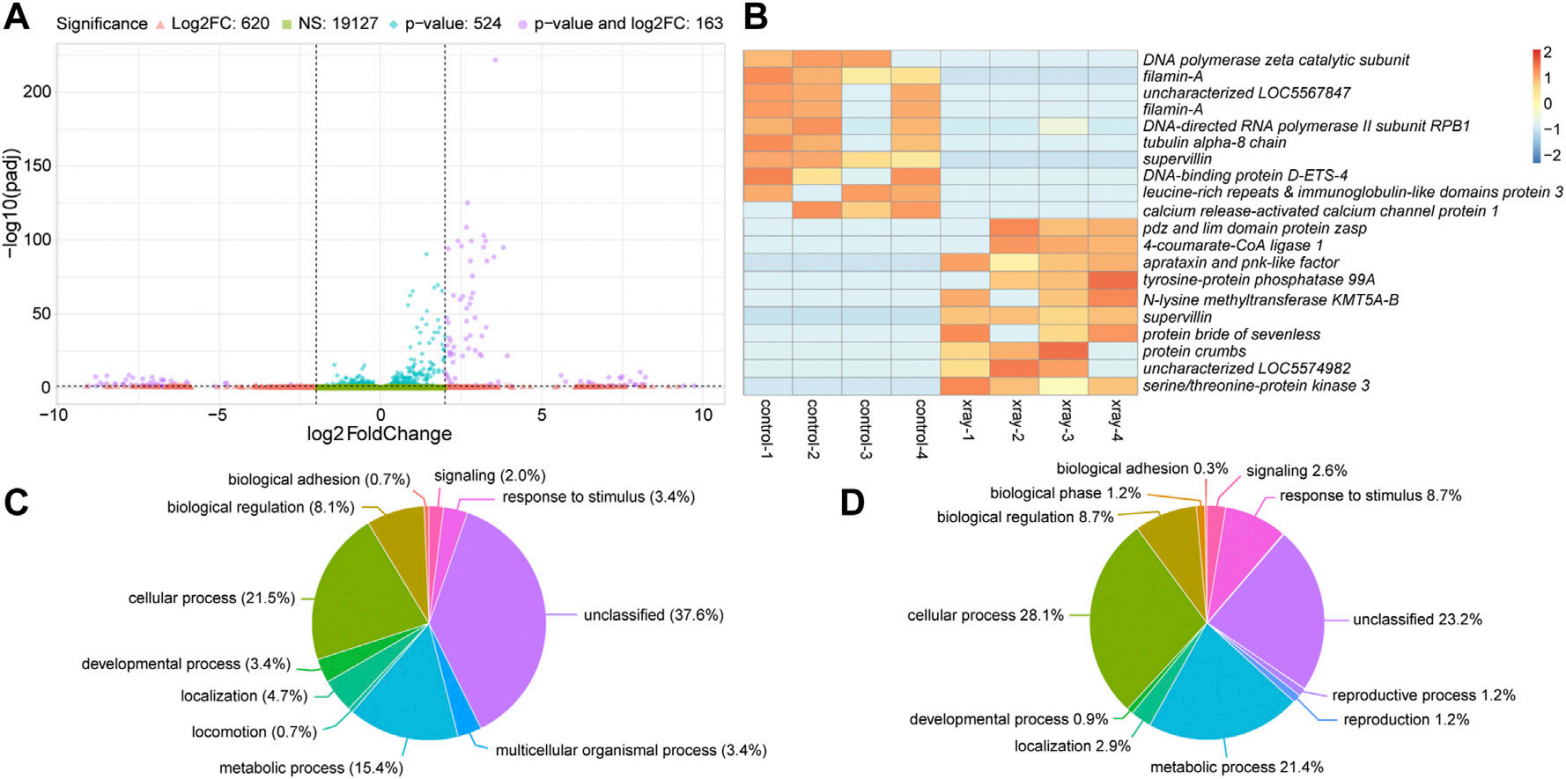
Differential gene expression between water-fed non-irradiated (control) and irradiated (x-ray) mosquitoes. **(A)**. Volcano plot illustrating the differential expression patterns between transcripts with elevated expression in non-irradiated mosquitoes (negative values) and elevated expression in irradiated mosquitoes (positive values). See Figure 2 legend for detailed description of volcano plot. **(B)**. Heatmap showing the ten most significantly differentially expressed transcripts with at least four-fold increase in non-irradiated or irradiated mosquitoes. Each column represents a single biological replicate, and each row represents an individual transcript. **(C and D)**. Pie charts of gene ontology (GO) analyses of genes upregulated in water-fed non-irradiated mosquitoes relative to irradiated mosquitoes **(C)** and upregulated in irradiated mosquitoes relative to non-irradiated mosquitoes **(D)**. See Figure 2 legend for detailed description of these charts.

We next analyzed changes in gene expression between water-fed non-irradiated and water-fed irradiated mosquitoes. Of the 20,434 transcripts we detected in these two treatments, 19,127 were not significantly different and were expressed within four-fold difference of each other (Figure 3A). A further 620 were at least four-fold different between the two treatment groups, but not significantly different (Figure 3A). The remaining 687 transcripts (compiled in Supplementary Table S3) were significantly differentially expressed (*p* ≤ 0.05), and 163 of these were also greater than four-fold differentially expressed between the two treatments (Figure 3A). The top ten upregulated transcripts in water-fed non-irradiated and irradiated treatments are shown in Figure 3B (a full heatmap of all differentially expressed transcripts is presented in Supplementary Figure S2). We performed this same analysis on ethanol-fed mosquitoes and observed a similar number of significantly differentially expressed genes between irradiated and non-irradiated ethanol-fed mosquitoes (Supplementary Table S4, Supplementary Figure S3).

Gene ontology analysis revealed changes in the relative enrichment of genes after irradiation, including increases in enrichment of genes associated with the GO biological process terms, cellular process, metabolic process, and response to stimulus relative to non-irradiated mosquitoes, and enrichment of genes associated with reproduction and reproductive process which were not enriched in non-irradiated controls (Figures 3C, D). Analyzing enrichment of GO biological process child terms revealed a significant enrichment of genes associated with response to ionizing radiation, DNA binding, telomere maintenance, and DNA repair in irradiated mosquitoes (Table 2). We also observed a set of enriched GO terms in our water-fed non-irradiated mosquitoes, which were associated with general biological processes such as metabolism and other cellular processes (Table 2). We performed this same analysis on ethanol-fed irradiated mosquitoes and observed a similar pattern in the gene ontology profile after ethanol feeding as well (Supplementary Table S5).

**TABLE 2:** Top ten gene ontology (GO) biological process terms identified in differentially expressed genes between water-fed non-irradiated and irradiated male *Ae. aegypti*. Gene ontology terms represent both parent and child terms enriched with genes either upregulated in irradiated mosquitoes relative to non-irradiated mosquitoes or upregulated in non-irradiated mosquitoes relative to irradiated mosquitoes.

Increased in water-fed irradiated					
Panther GO-Slim biological process	Genes in category	Genes in dataset	Fold enrichment	*p*-value	FDR
Response to ionizing radiation (GO: 0010212)	4	3	36.02	2.77E-04	2.74E-02
Cellular response to abiotic stimulus (GO: 0071214)	9	5	26.69	5.88E-06	1.02E-03
Cellular response to environmental stimulus (GO: 0104004)	9	5	26.69	5.88E-06	9.41E-04
Interstrand cross-link repair (GO: 0036297)	8	4	24.02	7.38E-05	8.08E-03
DNA strand elongation involved in DNA replication (GO: 0006271)	12	5	20.01	1.73E-05	2.57E-03
Response to radiation (GO: 0009314)	14	5	17.15	3.14E-05	4.36E-03
Nucleotide-excision repair (GO: 0006289)	20	7	16.81	8.57E-07	2.23E-04
Telomere maintenance (GO: 0000723)	17	5	14.13	6.76E-05	8.28E-03
Telomere organization (GO: 0032200)	17	5	14.13	6.76E-05	7.82E-03
Double-strand break repair (GO: 0006302)	55	11	9.61	8.39E-08	3.49E-05

Increased in Water-fed non-irradiated					
Panther GO-Slim Biological Process	Genes in category	Genes in dataset	Fold enrichment	*p*-value	FDR
Unclassified (UNCLASSIFIED)	6,822	56	0.64	3.94E-06	2.74E-03
Biological_process (GO: 0008150)	5,712	38	0.52	5.72E-08	1.19E-04
Metabolic process (GO: 0008152)	3,476	23	0.51	1.24E-04	4.31E-02
Organic substance metabolic process (GO: 0071704)	3,327	22	0.51	1.48E-04	3.85E-02
Cellular metabolic process (GO: 0044237)	3,111	20	0.5	2.02E-04	4.66E-02
Cellular process (GO: 0009987)	4,979	32	0.5	3.23E-07	3.36E-04
Nitrogen compound metabolic process (GO: 0006807)	2,991	18	0.47	1.09E-04	4.52E-02
Macromolecule metabolic process (GO: 0043170)	2,744	16	0.45	1.42E-04	4.21E-02
Primary metabolic process (GO: 0044238)	3,124	18	0.45	3.00E-05	1.56E-02
Biological regulation (GO: 0065007)	2,268	12	0.41	2.20E-04	3.81E-02

### 3.4 Differential response to irradiation between water-fed and ethanol-treated mosquitoes

**FIGURE 4:**
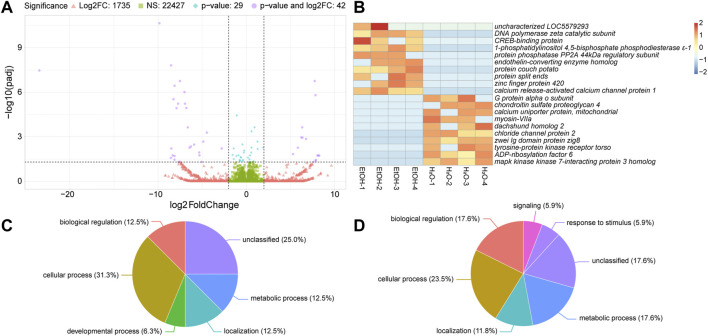
Differential gene expression between ethanol (EtOH) and water-fed irradiated mosquitoes. **(A)**. Volcano plot illustrating the differential expression patterns between transcripts with elevated expression in EtOH-fed mosquitoes (negative values) and elevated expression in water-fed mosquitoes (positive values). See Figure 2 legend for detailed description of volcano plot. **(B)**. Heatmaps showing the ten most significantly differentially expressed transcripts with at least four-fold increase in ethanol-fed mosquitoes or water-fed mosquitoes. Each column represents a single biological replicate, and each row represents an individual transcript. **(C and D)**. Pie charts of gene ontology (GO) analyses of genes upregulated in water-fed mosquitoes relative to ethanol-fed mosquitoes **(C)** and upregulated in ethanol-fed mosquitoes relative to water-fed mosquitoes **(D)**. See Figure 2 legend for detailed description of these charts.

We next analyzed differences in gene expression between ethanol-fed and water-fed irradiated mosquitoes. Of the 24,233 transcripts we detected in these two treatments, 22,427 were not significantly different, and were expressed within four-fold difference of each other (Figure 4A). Another 1,735 transcripts were expressed at least four-fold differentially between the two treatments but were not significantly different (Figure 4A). The remaining 71 transcripts (compiled in Supplementary Table S6) were significantly differentially expressed (*p* ≤ 0.05), and 42 of these were also greater than four-fold differentially expressed (Figure 4A). The top ten upregulated transcripts in ethanol-fed irradiated and water-fed irradiated treatments are shown in Figure 4B (a full heatmap of all differentially expressed transcripts is presented in Supplementary Figure S4).

We performed GO analysis on the differentially expressed genes (Figures 4C, D) and observed increases in the enrichment of genes associated with biological regulation and metabolic process in ethanol-fed mosquitoes relative to water-fed mosquitoes. Additionally, we observed enrichment of genes associated with signaling and response to stimulus in ethanol-fed mosquitoes that were not enriched in water-fed mosquitoes (Figures 4C, D). However, with so few differentially expressed genes between water-fed and ethanol-fed mosquitoes, GO analysis provided minimal GO terms for this comparison, so we chose to describe specific genes with differential expression between ethanol and water-fed males (Table 3).

**TABLE 3:** Top ten genes with significantly different gene expression between ethanol-fed and water-fed irradiated male *Ae. aegypti*.

Increased in ethanol-fed				
Vectorbase ID	Entrez gene ID	Name	log_2_Fold change	padj
AAEL019500-RC	5579293	uncharacterized LOC5579293	**23.44986863**	3.41E-08
AAEL009851-RD	5572510	DNA polymerase zeta catalytic subunit	**9.849643445**	2.25E-11
AAEL017391-RB	23687811	CREB-binding protein	**8.542393513**	0.026499231
AAEL027150-RB	5570306	1-phosphatidylinositol 4,5-bisphosphate phosphodiesterase epsilon-1	**8.51970371**	1.55E-08
AAEL019700-RG	5579206	protein phosphatase PP2A 55 kDa regulatory subunit	**8.497842068**	0.006132925
AAEL009895-RI	5572559	endothelin-converting enzyme homolog	**8.345798548**	0.017870446
AAEL028101-RD	110679566	protein couch potato	**8.251874306**	2.94E-06
AAEL009430-RE	5571946	protein split ends	**8.136317817**	0.019451136
AAEL019833-RAS	5566385	zinc finger protein 420	**8.133763862**	0.03500381
AAEL023441-RA	5579438	calcium release-activated calcium channel protein 1	**8.126910153**	3.62E-07

Increased in water-fed				
Vectorbase ID	Entrez Gene ID	Name	log_2_Fold Change	padj
AAEL008641-RM	5570867	G protein alpha o subunit	**8.254660824**	0.018514217
AAEL001803-RC	5572437	chondroitin sulfate proteoglycan 4	**8.158451667**	0.018174806
AAEL013565-RF	5578225	calcium uniporter protein, mitochondrial	**8.057410102**	0.018174806
AAEL011436-RB	5574835	myosin-VIIa	**7.870284086**	0.035944029
AAEL002684-RD	5575667	dachshund homolog 2	**7.856591204**	0.042335746
AAEL005950-RG	5567294	chloride channel protein 2	**7.83256856**	1.56E-06
AAEL007129-RF	5568775	zwei Ig domain protein zig-8	**7.742963193**	1.75E-07
AAEL018341-RO	5566523	tyrosine-protein kinase receptor torso	**7.601854441**	0.04942158
AAEL004913-RI	5565647	ADP-ribosylation factor 6	**6.765441146**	0.001394009
AAEL026170-RM	110678810	mitogen-activated protein kinase kinase kinase 7-interacting protein 3 homolog	**6.366999229**	0.001136261

log_2_Fold Change values represent the log_2_-normalized fold change in gene expression between ethanol-fed and water-fed mosquitoes.

### 3.5 Identification of potential “radioprotector proteins”

We were interested in identifying ethanol-induced transcripts that may be useful for “priming” mosquitoes to respond more favorably to irradiation. To that end, in addition to comparing feeding and irradiation treatments, we also cross-referenced transcripts that were significantly upregulated in ethanol-fed non-irradiated mosquitoes to the transcripts that were significantly upregulated in water-fed irradiated mosquitoes. We identified a small set of seven transcripts that fit this pattern (Table 4). These transcripts code for an enzyme involved in biosynthesis of antioxidant compounds (*4-coumarate-CoA ligase 1*), a p53-binding protein (*zinc finger protein 420*), two RNA-binding proteins (*heterogeneous nuclear ribonucleoprotein L*, and *hrp65*), a probable cytochrome P450 (*probable cytochrome P450 6d4*), and two enzymes involved in lipid metabolism (*probable peroxisomal acyl-coenzyme A oxidase 1*, and *1-acyl-sn-glycerol-3-phosphate acyltransferase delta*) (Table 4).

**TABLE 4:** Genes that may be involved in ethanol priming of mosquitoes for radiation treatment. These transcripts were identified to be significantly upregulated in ethanol-fed non-irradiated mosquitoes relative to water-fed non-irradiated mosquitoes and in water-fed mosquitoes after irradiation relative to non-irradiated water-fed mosquitoes, or significantly downregulated in ethanol-fed non-irradiated mosquitoes relative to water-fed non-irradiated mosquitoes and in water-fed mosquitoes after irradiation relative to non-irradiated water-fed mosquitoes.

Upregulated transcripts		
Vectorbase ID	Entrez gene ID	Name
AAEL002658-RC	5575571	*4-coumarate-CoA ligase 1*
AAEL019833-RAQ	5566385	*zinc finger protein 420*
AAEL025268-RK	5572228	*heterogeneous nuclear ribonucleoprotein L*
AAEL017116-RC	23687536	*hrp65 protein*
AAEL009129-RB	5571544	*probable cytochrome P450 6d4*
AAEL000735-RA	5566281	*probable peroxisomal acyl-coenzyme A oxidase 1*
AAEL022867-RA	5573302	*1-acyl-sn-glycerol-3-phosphate acyltransferase delta*

Downregulated transcripts		
Vectorbase ID	Entrez Gene ID	Name
AAEL026946-RC	110678647	*C-myc promoter-binding protein*
AAEL008680-RB	5570952	*ubiquitin-related modifier 1 homolog*
AAEL014551-RB	5564668	*pancreatic lipase-related protein 2*
AAEL003203-RC	5577524	*acyl-CoA delta(11) desaturase*
AAEL008543-RB	5570780	*cdc42 homolog*
AAEL019567-RN	5568502	*ensconsin*
AAEL012513-RU	5576419	*calcium-binding protein E63-1*
AAEL023148-RE	5572167	*filamin-A*
AAEL005147-RC	5566047	*DNA-binding protein D-ETS-4*

In addition to transcripts which were significantly upregulated in both ethanol-fed non-irradiated and water-fed irradiated mosquitoes, we identified a set of nine transcripts that were significantly downregulated in ethanol-fed non-irradiated mosquitoes and in water-fed irradiated mosquitoes relative to water-fed non-irradiated mosquitoes (Table 4). Because reduction of expression of these transcripts may also provide a radioprotective effect, we included them in our analysis.

## 4 Discussion

Sterile Insect Technique (SIT) is a mechanism of biological pest control with a successful history of eliminating dipteran insect pests (Bushland et al., 1955; Knipling, 1959; 1960; Vargas-Terán et al., 1994; Vreysen et al., 2000; Ant et al., 2012; Hyseni et al., 2012; Vargas-Terán et al., 2021). The use of ionizing radiation is a viable mechanism of producing large amounts of sterile male insects for mass release, but issues of fitness reduction due to deleterious effects of radiation exposure must be addressed during implementation of a SIT program (Parker et al., 2021). Ethanol consumption prior to exposure has been shown to provide protective effects against the cytotoxic effects of ionizing radiation in various model systems, including mosquitoes (Puddey et al., 1998; Wu and Cederbaum, 2003; Albano, 2006; Rodriguez et al., 2013). We hypothesize that ethanol exposure triggers a hormesis effect in male mosquitoes. Hormesis is defined as a biphasic dose-response to a stressor where the low exposure results in a beneficial effect while the high exposure is toxic (Calabrese and Baldwin, 2003; Mattson, 2008). We therefore focused our analysis on detoxifying enzymes and metabolic pathways, which could prime ethanol-treated mosquitoes to better repair and detoxify damaged cellular machinery and toxic byproducts after exposure to ionizing radiation.

### 4.1 Irradiation triggers a robust transcriptional response

Ionizing radiation is well-known to cause damage to double-stranded DNA, and lead to the production of cytotoxic ROS (Riley, 1994). In addition to attacking nucleic acids, ROS can also damage lipids in a process called lipid peroxidation (Yin et al., 2011) which can be particularly harmful to the cell if large portions of membrane lipids are damaged. All organisms have mechanisms to repair damage to their DNA, and most possess metabolic pathways to eliminate excessive ROS. Therefore, it was not surprising to see increased expression of many genes associated with abiotic stress responses and DNA binding and repair in mosquitoes exposed to 50 Gy of ionizing radiation regardless of feeding treatment (Table 2; Figure 3).

### 4.2 Minute effects of dietary ethanol on male mosquito transcriptomes

We were surprised to identify just a small number of genes associated with ethanol-priming treatment in male mosquitoes. We will focus our discussion in this section on the genes with the highest expression in ethanol-fed non-irradiated mosquitoes relative to water-fed non-irradiated mosquitoes, as these genes are more likely to influence survival in response to irradiation. We acknowledge that other mechanisms that do not involve gene transcription may be important for enhancing survival in ethanol-treated males after irradiation. Future studies of this problem will therefore include metabolomics and proteomics analyses.

In ethanol-fed non-irradiated mosquitoes, we identified a set of genes that are associated with cellular processes that may be useful for mitigating cytotoxic effects of ethanol. The most significantly increased gene in ethanol-fed mosquitoes was *marf*, an ortholog of mammalian *mitofusin 2* (*mfn2*) which is a GTPase that functions as a mitochondrial fusion factor (Chen et al., 2003). Global knockdown of *marf* in *D. melanogaster* larvae caused lethality prior to pupation (Dorn et al., 2011), demonstrating the necessity of maintenance of mitochondrial structure to survival. Ablation of *mfn2* in mouse cerebellum led to irregular development of Purkinje cells, and abnormal dendrite morphology (Chen et al., 2007). We propose that increased expression of this gene in ethanol-fed mosquitoes may be involved in mitigating mitochondrial fission and apoptosis, particularly to protect fat body cells from mitochondrial fragmentation during the build-up of toxic byproducts of ethanol detoxification, and to promote neuronal survival in response to ethanol toxicity.

The enzyme, *4-coumarate-CoA ligase*, is best classified in plants, where it serves as a metabolic inflection point to utilize cinnamic acid-derived molecules for the synthesis of lignin and a variety of secondary metabolites including antioxidants and other stress-induced molecules (Knobloch and Hahlbrock, 1975; Liu et al., 2017). We propose that this enzyme may be used to generate antioxidant molecules in response to oxidative stress generated by ethanol catabolism, which can then be useful for detoxifying ROS generated by radiation damage. Little is known about the function of *4-coumarate-CoA ligase* in animals, but phylogenetic analysis has grouped plant 4-coumarate-CoA ligase enzymes with a unique family of insect acyl-CoA synthetases including firefly luciferase (Oba et al., 2005). Because the functions of this enzyme are best classified in plants, metabolite profiling in response to *4-coumarate-CoA ligase* RNAi knockdown, or CRISPR/Cas-9 mediated knockout will provide valuable insights into what role this enzyme may play in antioxidant or lipid metabolism in *Ae. Aegypti*.

The RNA-binding protein, *heterogeneous nuclear ribonucleoprotein L* (*hnrnpl*), is associated with alternative mRNA splicing (Hung et al., 2008), with implicated roles in regulation of cell death and proliferation by regulating alternative splicing of caspase 9 (Goehe et al., 2010; Gu et al., 2020) and by forming a complex with p53, thereby regulating p53-mediated apoptosis (Gu et al., 2020). Research in human cells identified *park7*, a gene associated with protection from oxidative stress (Taira et al., 2004; Clements et al., 2006; Guzman et al., 2010), as a target for alternative splicing by *hnrnpl* (Hung et al., 2008). Additional work in mammalian cells demonstrated a role for *hnrnpl* in recruiting DNA break repair proteins to damaged DNA in cancer cells (Hu et al., 2019), indicating that this protein may also provide protection through activation of DNA damage response pathways.

We also identified *zinc finger protein 420* (also named *atm and p53-associated kznf protein*, or *apak*), which codes for a p53-binding protein that negatively regulates p53-mediated apoptosis (Tian et al., 2009). Overexpression of this gene in ethanol-fed mosquitoes may provide protection by inhibiting cell death in response to toxic effects of ethanol consumption. Interestingly, while this gene was significantly upregulated in irradiated water-fed mosquitoes relative to non-irradiated water fed controls (Table 2), it was significantly elevated in ethanol-fed mosquitoes relative to water-fed mosquitoes in both non-irradiated and irradiated groups (Tables 1, 3). Therefore, this gene may be a particularly important ethanol-induced survival gene. Loss-of-function experiments to determine how this gene may affect mosquito survival under normal, and stress conditions will help to understand how important *zinc finger protein 420* is to the prevention of cell death under different conditions and determine the effectiveness of *zinc finger protein 420* as a target for new mosquito control agents.

We identified genes associated with protein modification with increased expression in ethanol-fed non-irradiated mosquitoes relative to water-fed non-irradiated mosquitoes. One of these, *polypeptide N-acetylgalactosaminyltransferase 5* belongs to a family of glycotransferases that participate in glycosylation of extracellular matrix (ECM) protein components (Kato et al., 2021). The other gene, *aminopeptidase N* encodes a membrane-localized enzyme that removes amino acids from the N-terminal of proteins (Turner, 2013).

Several cytoskeleton-associated proteins were elevated in response to ethanol feeding. The *filamin b* gene (*flnb*) encodes an actin-binding protein that can cross-link actin filaments and link actin filaments to membrane-bound proteins (Nakamura et al., 2011). In addition to its actin-binding functions, FlnB has also been demonstrated to play a role in alternative splicing of apoptosis-related transcripts in HeLa cell death (Ma et al., 2020), and pro-apoptotic effects as a part of the type I interferon signaling pathway as assayed in mammalian cells (Jeon et al., 2008; Whitmarsh, 2009). Tubulin polyglutamylase TTLL5 (TTLL5) is a microtubule associated protein that glutamylates alpha tubulin subunits. Polyglutamylated tubulin is associated with more stable MTs, and polyglutamylated MTs are commonly found in nervous tissues (Yu et al., 2015). We propose that increased *ttll5* expression after ethanol treatment may serve to stabilize nerve cell morphology, particularly axon morphology in response to the cytotoxic effects of ethanol exposure.

We also identified genes associated with synaptic transmission. One of these, *syntaxin-binding protein 5* is a protein involved in regulation of synaptic vesicle fusion and neurotransmitter release (Fujita et al., 1998). We suggest that increased expression in ethanol-fed mosquitoes is to maintain normal nervous signaling in response to ethanol exposure. The other gene, *5-hydroxytryptamine receptor 2B* is a G-protein coupled serotonin receptor.

Taken together, these ten highly upregulated genes in ethanol-fed non-irradiated mosquitoes represent a set of genes/proteins that likely play protective roles against the toxic byproducts of ethanol metabolism in the fat body, and in the maintenance of proper neural function in response to chronic ethanol consumption. These functions are likely also important for ameliorating the cytotoxic effects of ionizing radiation, making these genes interesting high-value candidates for further study.

### 4.3 Possible radioprotective priming genes

Damage done to DNA in replicating cells, such as the gametes of male mosquitoes, will lead to sterilization, which is the backbone of the principle behind radiation-based SIT programs. However, the radiation dose must not be so high as to cause severe radiation sickness and reduce the fitness of irradiated males to the point where they can no longer compete with wild males for mating. Therefore, we searched our data for genes that may maximize male fitness after exposure to a sterilizing dose of ionizing radiation.

Even though relatively few genes were significantly differentially expressed between ethanol-fed and water-fed mosquitoes, we still identified a set of genes with significantly induced or reduced expression in ethanol-fed males that may play a role in priming male mosquitoes for better fitness in response to irradiation (Table 4). We propose that these are the most important candidate priming genes, due to their significantly different expression in ethanol-fed non-irradiated mosquitoes relative to water-fed non-irradiated mosquitoes, and in water-fed irradiated mosquitoes relative to water-fed non-irradiated mosquitoes.

We have previously discussed three of these potential priming genes already, as they were among the top 10 most upregulated genes in ethanol-fed non-irradiated mosquitoes relative to water-fed mosquitoes. Two of these genes are *4-coumarate-CoA ligase*, and *zinc finger protein 420*. It is likely that increased *4-coumarate-CoA ligase* expression prior to radiation treatment primes cells by having elevated levels of antioxidant molecules present in cells to scavenge ROS produced by ionizing radiation. Priming by *zinc finger protein 420* expression likely protects against radiation by reducing available p53 at the time of irradiation, thereby reducing the number of cells that undergo apoptosis. Increased cell survival after irradiation should increase organismal fitness in response to radiation treatment.

Two RNA-binding proteins also are potential priming genes as well. The first is *hnrnpl*, which was discussed previously. Priming of this gene by ethanol consumption likely protects against cytotoxic effects of radiation by regulating splicing of apoptotic factors and ROS detoxifying enzymes, and by priming DNA damage repair proteins in advance of irradiation. The other RNA-binding protein, *hrp65*, has been classified as an actin-binding protein in the dipteran *Chironomus tentans* (Miralles and Visa, 2001) that is thought to play an important role in RNA Polymerase II-mediated transcription (Percipalle et al., 2003; Sjölinder et al., 2005). Increased expression of *hrp65* may prime ethanol-fed mosquitoes to survive radiation by allowing for a more rapid initiation of expression of radioprotective genes.

The function of the cytochrome P450 gene, *probable cytochrome p450 6d4* (*cyp6d4*) is not well classified. In insects, cytochrome P450 family proteins are typically involved in metabolizing hormones and detoxifying exogenous compounds such as insecticides (Feyereisen, 1999). This *probable cyp6d4* enzyme does not seem to play a role in elimination of insecticides in *Drosophila* (Hardstone et al., 2006), but it is possible that it may detoxify other damaging molecules formed by ethanol exposure and/or ionizing radiation, such as toxic byproducts of lipid peroxidation caused by ROS. Paradoxically, cytochrome P450 enzymes often produce ROS as a by-product of their detoxification activities (Veith and Moorthy, 2018), which should cause increased oxidative stress in ethanol-fed and irradiated mosquitoes. There is some evidence that ROS can provide beneficial effects to cells when generated at low levels (Shields et al., 2021), so it is possible that ROS generation in ethanol-fed mosquitoes may trigger protective effects that are carried over after irradiation. However, it is more likely that ROS produced by *probable cyp6d4* activity are detoxified by antioxidants produced by increased *4-coumarate-CoA ligase* expression, and activities other antioxidant synthetic enzymes.

The final two upregulated priming genes we identified are associated with lipid metabolism. The first of these two genes is *probable peroxisomal acyl-coenzyme A oxidase 1*, which is involved in peroxisomal fatty acid beta oxidation (Aoyama et al., 1994). The second gene we identified is *1-acyl-sn-glycerol-3-phosphate acyltransferase delta*, which codes for an enzyme involved in phospholipid synthesis (Vance, 2002). Interestingly, we also identified two lipid-associated transcripts which were downregulated after ethanol feeding and after irradiation of water-fed mosquitoes, indicating that their downregulation may have a radioprotective effect. These two transcripts are *pancreatic lipase-related protein 2* and *acyl-CoA delta(11) desaturase*. We propose that differential expression of these genes may be used to help manage peroxidized triglycerides and to replenish membrane lipids that have been damaged due to lipid peroxidation.

Other potential genes that may provide a radioprotective effect through their downregulation include three cytoskeleton-associated transcripts, a calcium binding protein and three transcripts associated with gene expression processes. These were the actin binding protein, *filamin-A*, the microtubule associated protein, *ensconsin*, and *cdc42 homolog* which is the ortholog of Drosophila *cdc42*, a small GTPase that regulates the actin cytoskeleton (Leibfried et al., 2013). The calcium-binding protein *e63-1* transcript codes for a calcium-binding protein orthologous to the E63-1 protein in Drosophila. Expression of this protein is induced by 20-hydroxy ecdysone signaling in developing salivary glands (Andres and Thummel, 1995), but it has also been shown to be expressed in other larval tissues regardless of hormone regulation (Vaskova et al., 2000). The *DNA-binding protein d-ets-4* transcript encodes a transcription factor orthologous to *Drosophila ets98b*. Several ETS transcription factors have been demonstrated to limit Drosophila lifespan, but *ets98b* is not one of these (Dobson et al., 2019). Previous research in *Drosophila* larvae exposed to 0.1 Gy of gamma radiation showed an induction of *ets98b* in response to radiation (Kim et al., 2015). Interestingly, we saw the opposite response in expression of this gene in adult male *Ae. aegypti* after ethanol feeding, and in response to a 50 Gy dose of ionizing radiation. Two additional downregulated transcripts associated with gene expression processes identified were *C-myc promoter-binding protein* (*mbp-*1) and *ubiquitin-related modifier 1* (*urm1*). The first of these transcripts, *mbp-1* is known for its ability to negatively regulate the c-myc gene, a gene that is known to regulate cellular metabolism and increase cellular proliferation amidst cellular stress (Chaudhary and Miller, 1995). The second transcript, *urm1* is known for its role in tRNA modification and targeting protein regulation amidst various cellular stressors such as oxidative stress (Khoshnood et al., 2017).

Taken together, this small set of genes may prime male mosquitoes for better outcomes after irradiation through a variety of mechanisms. These include increased production of antioxidants, protection against apoptotic signaling, increasing levels of detoxifying enzymes, maintenance of cell structure, and altering lipid metabolism to protect against free radical generation and maintain the structure of cellular and organellar membranes. These genes cover a number of necessary responses to assist in cell survival after radiation exposure, and their altered expression prior to radiation exposure likely contributes to the observed increase in survival after radiation exposure that we have previously reported (Rodriguez et al., 2013). We acknowledge that a lack of independent validation of the expression patterns of the genes described in this study is a limitation that needs to be addressed in future experiments. Future studies to validate the expression levels of these genes and the tissue and organ specificity of expression of these genes using quantitative PCR will provide vital insight into the specific tissues that are most affected by radiation exposure and are most important for ameliorating the destructive effects of radiation. Additionally, studies designed to knock down or knock out these genes prior to irradiating male mosquitoes and observing changes in male fitness and mating success should be performed to fully elucidate any role these genes may play in protection from harmful effects of ionizing radiation.

## 5 Conclusion

Ionizing radiation has profoundly detrimental effects on cells, as it causes damage to nucleic acids and production of ROS. However, these effects can be implemented into beneficial programs such as radiation therapy and SIT programs. To provide the most successful outcomes of treatment with ionizing radiation, minimization of deleterious effects to non-targeted cells is necessary. In this study we have used Illumina RNA-seq to identify genes induced by consumption of ethanol that likely have radioprotective effects in male *Ae. aegypti* exposed to a sterilizing dose of ionizing radiation. This set of “priming” genes will inform the design of future experiments to determine their roles in protection from ionizing radiation. A deeper understanding of how these genes and their encoded proteins function will prove useful for any program utilizing ionizing radiation that needs to mitigate harmful off-target effects.

## Data Availability

The datasets presented in this study can be found in online repositories. The names of the repository/repositories and accession number (s) can be found below: https://www.ncbi.nlm.nih.gov/bioproject/?term=PRJNA895439.

## Supplementary material

Supplementary Table S1 Sequencing statistics for Illumina RNA-seq runs by Azenta.

Supplementary Table S2 Table of all significantly differentially expressed transcripts between ethanol-fed and water-fed non-irradiated mosquitoes. Transcript counts in TPM are given for each sample, and the log_2_-fold change was used to separate transcripts into groups with either increased expression in ethanol-fed mosquitoes (negative log_2_-fold change) or increased expression in water-fed mosquitoes (positive log_2_-fold change).

Supplementary Table S3 Table of all significantly differentially expressed transcripts between water-fed non-irradiated and irradiated mosquitoes. Transcript counts in TPM are given for each sample, and the log_2_-fold change was used to separate transcripts into groups with either increased expression in irradiated mosquitoes (positive log_2_-fold change) or increased expression in non-irradiated mosquitoes (negative log_2_-fold change).

Supplementary Table S4 Table of all significantly differentially expressed transcripts between ethanol-fed non-irradiated and irradiated mosquitoes. Transcript counts in TPM are given for each sample, and the log_2_-fold change was used to separate transcripts into groups with either increased expression in irradiated mosquitoes (positive log_2_-fold change) or increased expression in non-irradiated mosquitoes (negative log_2_-fold change).

Supplementary Table S5 Top ten gene ontology (GO) biological process terms identified in differentially expressed genes between ethanol-fed non-irradiated and irradiated male Ae. aegypti. Gene ontology terms represent both parent and child terms enriched with genes either upregulated in irradiated mosquitoes relative to non-irradiated mosquitoes or upregulated in non-irradiated mosquitoes relative to irradiated mosquitoes.

Supplementary Table S6 Table of all significantly differentially expressed transcripts between ethanol-fed and water-fed irradiated mosquitoes. Transcript counts in TPM are given for each sample, and the log_2_-fold change was used to separate transcripts into groups with either increased expression in ethanol-fed mosquitoes (negative log_2_-fold change) or increased expression in water-fed mosquitoes (positive log_2_-fold change).

Supplementary Figure S1 Heatmap of all significantly differentially expressed transcripts between ethanol-fed and water-fed non-irradiated (control) mosquitoes. A heatmap of log_2_ scaled, normalized TPM counts for all up and down-regulated genes that are significantly differentially expressed (p-adjusted value cutoff of 0.05) between ethanol-fed and water-fed non-irradiated mosquitoes.

Supplementary Figure S2 Heatmap of all significantly differentially expressed transcripts between water-fed non-irradiated (control) and irradiated mosquitoes. A heatmap of log_2_ scaled, normalized TPM counts for all up and down-regulated genes that are significantly differentially expressed (p-adjusted value cutoff of 0.05) between water-fed non-irradiated and water-fed irradiated mosquitoes.

Supplementary Figure S3 Heatmap of all significantly differentially expressed transcripts between ethanol-fed non-irradiated (control) and irradiated mosquitoes. A heatmap of log2 scaled, normalized TPM counts for all up and down-regulated genes that are significantly differentially expressed (p-adjusted value cutoff of 0.05) between ethanol-fed non-irradiated and ethanol-fed irradiated mosquitoes.

Supplementary Figure S4 Heatmap of all significantly differentially expressed transcripts between ethanol-fed and water-fed irradiated mosquitoes. A heatmap of log_2_ scaled, normalized TPM counts for all up and down-regulated genes that are significantly differentially expressed (p-adjusted value cutoff of 0.05) between ethanol-fed and water-fed irradiated mosquitoes.
