# Supplementary material for: Transcriptomics analysis of ethanol treatment of male Aedes aegypti reveals a small set of putative radioprotective genes
Source: Front Physiol. 2023 Jan 30;14:1120408. doi: 10.3389/fphys.2023.1120408 (PMC9922702; doi:10.3389/fphys.2023.1120408)

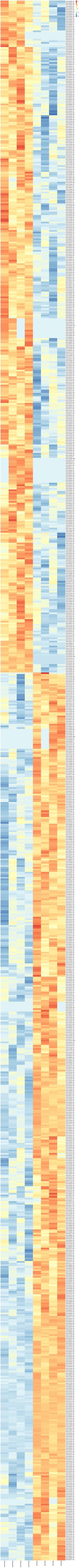

Supplement: Supplementary file 6 [file Image3.PDF]
